# Fluxion Dilutions

**Published:** 1881-08

**Authors:** Eldridge C. Price

**Affiliations:** Baltimore, Md.


### FLUXION DILUTIONS.

ELDRIDGE C. PRICE, M. D., BALTIMORE, MD.

THE ability to perfectly express an idea is a gift. The ability to perfectly understand an idea is also a gift. Few men possess either, fewer possess both.

My friend, Dr. Wm. Jefferson Guernsey, criticises my article on Fluxion Dilutions, that appeared in the *Hahnemannian Monthly*, a few months since.

His criticism is unjust in that he has not read my article carefully, and considered sufficiently the points I really have claimed. His impression of my belief is obtained from a superficial view of the subject. From Dr. Guernsey's article, the reader is impressed that I am an exclusive low-potency advocate—a materialist. Did I not highly esteem my friend, I should hesitate to trouble myself with the correction of this error, because the opinion was not formed from the article criticised, or from anything else I may have written.

I am of the class called dynamists, and occupy a middle ground between the materialists and the altissimo-dilutionists.

The principles guiding the dynamists, are those laid down by Hahnemann in his Organon. The position I hold is the result of mature deliberation, founded upon unrefuted theory and experience, and my written opinion is simply the offspring of my honest convictions.

In my article on the fluxion dilutions, there is nothing inconsistent with my beliefs, and, therefore, I do not retract one word I have written.

I quote from the article in question: "A drug diluted to any degree we may choose, is therefore not a potency, unless friction has been applied in each degree of the process." I insist upon this as the basis of potency belief of every true disciple of Hahnemann.

That potency and dilution are not synonymous, is a fact not appreciated by all. This seems to be the mistake Dr. Guernsey has made.

To make a *potency*, friction is a necessary factor; to make a *dilution* it is not.

The preparations of Swan and Fincke are, therefore, dilutions, because they lack the friction element. They nevertheless claim to be potencies; they are not; therefore, they are shams.

Dr. Guernsey says that I "cannot prove that a very high potency does not exist."

This I can and have proved. My statement reads: "I do not believe a single genuine 100,000th Hahnemannian potency has ever been made."

I did not say a Fincke *dilution*, but a Hahnemannian *potency* If Dr. Guernsey, or any one else, knows of a *bona fide* 100,000th

Hahnemannian *potency*, I shall be pleased to read an account of its preparation.

Again, Dr. Guernsey states that I "savagely" declare that these fluxion dilutions "will not or do not act." This, like his opinion about my potency belief, is obtained from a source of which I know not. But let me quote my savage declaration: "The reported cures by very high fluxion dilutions are, therefore, really produced by either, 1st, the patient's imagination, or 2d, infinitesimal particles of drug substances that happen not to have been washed out of the fluxion apparatus, and are analogous to particles found in the low Hahnemannian preparations."

This is not a denial of the ability of some of these preparations to act; on the contrary, I acknowledge the *possibility* of cures by the fluxion dilutions. But these cures are not made by *bona fide high potencies;* they are produced by those particles of diluted drug substance which have remained in the apparatus, and are really equivalent to cures made by the lower Hahnemannian preparations.

Obviously, then, the fluxion dilutions *may* act. Granted; but so may *bona fide* potencies.

Suppose the percentage of cures by the two classes is equal, we gain nothing by substituting these *dilutions* for our *potencies*. Why, then, should we force these preparations into our pharmacopœia, when we have genuine potencies that are what they claim to be, and that will produce all the curative results that it is possible to extract from these pseudo-potencies?

It is even desired to substitute these dilutions for potencies. To make so vital a change in pharmacology, some great advantage should result. Apropos, what are we to gain by accepting and using the fluxion dilutions?

That I may fully convince my friend, Dr. Guernsey, of my practical belief in high potencies, and that I really do use drugs above the "red, white or blue," and also to illustrate the fact that these potencies may produce cures that even the greatest fluxion enthusiast cannot excel with his choicest dilutions, I will quote a few cases from actual practice:

Miss L. came to me, after having suffered, for some weeks, with the following symptoms:—*Intense* pains, erratic in character, worse in warm room, better in open air and from motion. Despondency, tendency to cry without cause. I gave her *Puls.* 2 C, four powders, one daily at night on retiring. This prescription cured permanently within a week, a year having elapsed without a return.

C. W. Was called hurriedly to see a woman who had been suffering for some hours as follows:—Violent emesis; diarrhœa, stools thin and watery, accompanied with tenesmus and cramping pains in abdomen; coldness of extremities and cold sweat on forehead; also great weakness. The discharges were so frequent, and the weakness was so great, that she was unable to leave the vessel upon which she sat. Prescribed *Verat. alb.* 35th, a powder in half a glass of water, teaspoonful after each stool. She began to improve immediately after the first dose, the stools becoming less frequent, and in twelve hours she was perfectly cured.

B. F. S. called to see me, December 16th, 1880. He has for some years suffered more or less from the following symptoms:—Eruption of small pimples, which are at times painful, on face, back and shoulders. No perspiration about the face, but in its stead an oozing of oil from the sebaceous follicles. Flushes of heat into upper part of body, which aggravate the eruption. Undue fullness after eating, with occasional eructations of gas. Bowels inclined to constipation, with no desire to defecate. Coffee, pork and mince-meat disagree. *Silicea* 30th, a powder three times a day. This remedy was continued for some time, but the potency was changed to 2 C, and the frequency of dose diminished, until he took but one dose in a week. His condition improving, but the symptoms changing to indications for *Bry.* This remedy was given in the 2 C potency, sometimes two or three weeks elapsing between doses.

June 9th, 1881, he reports the eruption has almost entirely disappeared. The only part now affected is around the nose, and this is very slight. No other symptoms; he feels perfectly well. *Bry.* 2 C, one dose and S. L.

Mrs. S. Suffered for about four years with constipation; stools in narrow, ribbon-like strips. *Phos.* 35th, three or four powders in as many days; relieved within a week.

Miss C. Subject every fall and winter to attacks of bronchitis. I saw her in November, 1880. She complained of pain in chest, with oppression; tight, dry cough, worse at night and on motion. She had been sick about a month. I gave her a few powders of *Bry.* 35th, once or twice daily for a few days. Improvement began immediately, and within a week she was cured.

I have just heard of her robust health, which has continued uninterruptedly, notwithstanding her country residence and the unusually severe winter, in which she observed no especial precautions against contracting her usual attacks of bronchitis, but the first time for years she has remained free from them.

When my friend of "auld lang syne" re-reads my article in the *Hahnemannian Monthly*, and then has carefully read this sequel to it, I do not think he will continue to consider me either savage, uncharitable, a materialist, or an altissimist, but plainly and simply a truth-seeking homœopathist, a dynamist.

